# Crystal structure of mammalian acid sphingomyelinase

**DOI:** 10.1038/ncomms12196

**Published:** 2016-07-20

**Authors:** Alexei Gorelik, Katalin Illes, Leonhard X. Heinz, Giulio Superti-Furga, Bhushan Nagar

**Affiliations:** 1 Department of Biochemistry, McGill University, Montreal, Quebec, Canada H3G 0B1; 2 Groupe de Recherche Axé sur la Structure des Protéines, McGill University, Montreal, Quebec, Canada H3G 0B1; 3 CeMM Research Center for Molecular Medicine of the Austrian Academy of Sciences, 1090 Vienna, Austria; 4 Center for Physiology and Pharmacology, Medical University of Vienna, 1090 Vienna, Austria

## Abstract

Acid sphingomyelinase (ASMase, ASM, *SMPD1*) converts sphingomyelin into ceramide, modulating membrane properties and signal transduction. Inactivating mutations in ASMase cause Niemann–Pick disease, and its inhibition is also beneficial in models of depression and cancer. To gain a better understanding of this critical therapeutic target, we determined crystal structures of mammalian ASMase in various conformations. The catalytic domain adopts a calcineurin-like fold with two zinc ions and a hydrophobic track leading to the active site. Strikingly, the membrane interacting saposin domain assumes either a closed globular conformation independent from the catalytic domain, or an open conformation, which establishes an interface with the catalytic domain essential for activity. Structural mapping of Niemann–Pick mutations reveals that most of them likely destabilize the protein's fold. This study sheds light on the molecular mechanism of ASMase function, and provides a platform for the rational development of ASMase inhibitors and therapeutic use of recombinant ASMase.

## Abstract (web summary)

Mutations in acid sphingomyelinase result in toxic accumulation of sphingomyelin and cause Niemann-Pick disease. Here, the authors report structures of mammalian acid sphingomyelinase, which reveal details of the molecular mechanism of acid sphingomyelinase function and regulation.

## Graphical abstract

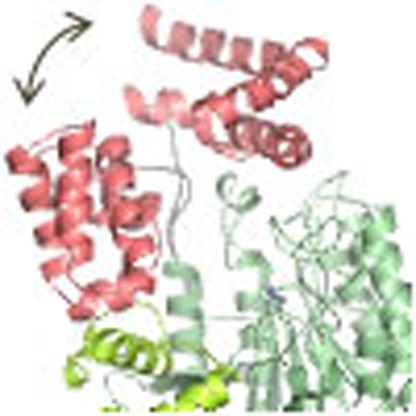

Acid sphingomyelinase (ASMase, ASM, *SMPD1*) is an enzyme found in lysosomes and in the extracellular space where it catalyses the conversion of sphingomyelin, a major component of membranes, into ceramide and phosphocholine^1^,^2^. Ceramide is a key signalling lipid that modulates membrane biophysical properties and is involved in numerous cellular processes as well as disease states. Hereditary mutations of ASMase result in the toxic accumulation of sphingomyelin and are the cause of the lysosomal storage disease Niemann–Pick type A, which is neuropathic and fatal, and type B, which causes visceral anomalies^3^. Clinical trials for treating this disease have been carried out using recombinant ASMase as enzyme replacement therapy^1^.

More recently, it was discovered that inhibition of ASMase activity mediates the effects of antidepressant drugs in hippocampal neurons^4^,^5^, selectively kills cancer cells by destabilizing their fragile lysosomes^6^,^7^, reduces inflammation associated with cystic fibrosis^8^, decreases atherosclerotic lesions^9^ and diminishes symptoms associated with Alzheimer's disease^10^. These findings have established ASMase as a critical therapeutic target. However, while its activity has been shown to be indirectly inhibited by certain antidepressants^4^ and other cationic amphiphilic drugs (CADs) that lead to its degradation, no direct inhibitor of ASMase is currently in medical use. Although the protein was characterized over 30 years ago, knowledge of its three-dimensional structure for aid in inhibitor development is still lacking.

ASMase consists of an N-terminal saposin domain and a C-terminal catalytic domain. The saposin domain is found in small lysosomal proteins that generally act in isolation by binding to or extracting lipids from the membrane for subsequent breakdown by hydrolytic enzymes, and the structures of several saposins have been determined^11^,^12^,^13^,^14^. Sequence analysis of the catalytic domain classifies it as a phosphoesterase enzyme and, recently, the crystal structure of the ASMase paralogue, acid sphingomyelinase-like phosphodiesterase 3A (SMPDL3A) was reported^15^,^16^, which provided some insight into the catalytic mechanism. However, SMPDL3A cannot cleave sphingomyelin and hydrolyzes nucleotides instead^17^. Moreover, it lacks a saposin domain, which limits its usefulness for understanding how ASMase works.

To gain insight into the molecular mechanism of ASMase function and the interplay between its domains, we determined crystal structures of the full-length protein from mouse that reveal a conformational flexibility of the saposin domain. In one conformation, the saposin domain adopts a globular closed form independent of the catalytic domain, while in the other conformation, it assumes an open V-shaped fold that establishes an extended interface with the catalytic domain. Activity assays demonstrate that this interface is indispensable for substrate hydrolysis.

## Results

### Overview

**Figure 1:**
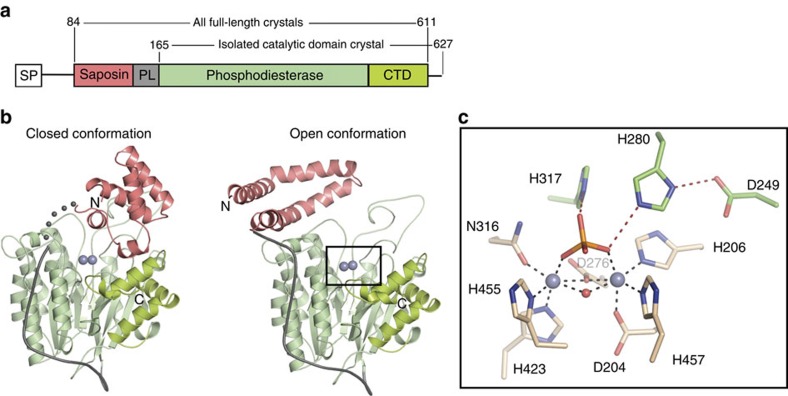
Structural overview of ASMase. (**a**) Domain organization of ASMase. SP, signal peptide; PL, proline-rich linker. (**b**) Closed and open ASMase coloured as in **a**. Consecutive grey spheres indicate the disordered linker between closed ASMase^sap^ and ASMase^cat^ in one molecule of the asymmetric unit. Boxed region magnified in **c** is not in the identical orientation. Glycans are omitted for clarity. Zincs are indicated as purple spheres. (**c**) ASMase active site. Zinc interactions are shown as dashes. Zinc–zinc distance=3.6 Å. Zinc–ligand distances range from 2.0 to 2.1 Å. Phosphate is coloured orange and red. Shown are zinc-interacting residues (beige) and residues important for leaving group protonation and substrate binding (green).

ASMase is synthesized and differentially modified post-translationally in the endoplasmic reticulum and Golgi to yield distinct lysosomal and secreted forms. Our crystallized construct (murine residues 84–611 corresponding to human residues 88–615, 88% identity to human ASMase; Fig. 1a) produced in insect cells most closely resembles the well-characterized lysosomal form with regards to glycosylation pattern, cleavage of a nine-residue C-terminal peptide that renders the enzyme fully active^18^,^19^, and N-terminal processing up to residue 84 (human residues 85–622)^20^. We determined several structures of ASMase (Table 1) in various forms including two distinct conformations in which the N-terminal saposin domain (ASMase^sap^) changes its fold and position relative to the catalytic domain (ASMase^cat^, Fig. 1b).

### Catalytic domain and active site

**Figure 2:**
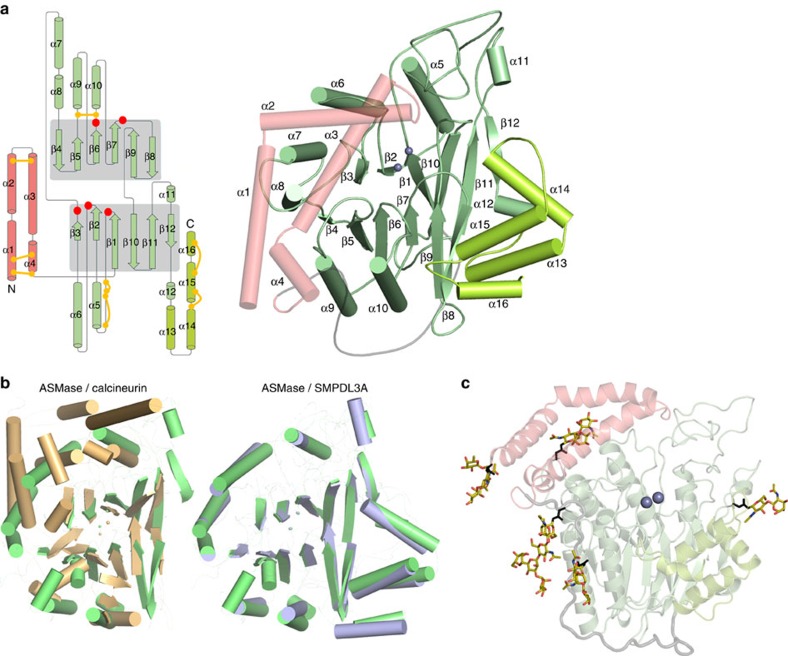
The ASMase fold. (**a**) Left: topology diagram of ASMase. The central β-sheets of the catalytic domain are highlighted with grey shading. Strands are represented by arrows and α-helices by cylinders. The topology of the open form of the saposin domain (pink) is shown; in the closed form, α-helix 3 is divided into two helices. Disulfide bonds are represented by yellow connectors. Red circles indicate regions of metal binding or catalytic residues. CTD helices are coloured lime green. Right: structure of ASMase with secondary structure elements labelled. (**b**) Comparison of the ASMase structure with related phosphodiesterases. The β-sandwich fold of the ASMase catalytic domain (green) is comparable to that of related phosphodiesterases, including calcineurin (PDB code 3LL8, overall sequence identity=10%, root mean squared deviation (r.m.s.d.)=1.9 Å for 40 corresponding α-carbons from the conserved β-strands only), displayed in gold. In addition, the catalytic and C-terminal domains of ASMase are conserved in SMPDL3A (PDB code 5FC1, overall sequence identity=29%, r.m.s.d.=0.9 Å for 281 corresponding α-carbons), which is displayed in blue. (**c**) Glycosylation sites (coloured sticks) on ASMase produced from insect cells. Asn N-glycan attachment residues are shown as black sticks.

We begin by describing ASMase^cat^, which was also crystallized in isolation. Its core, composed of two six-stranded mixed β-sheets surrounded by eight α-helices, belongs to the calcineurin-like phosphoesterase structural family (Pfam PF00149) and resembles recent structures of the ASMase-like protein, SMPDL3A (refs 15, 16) (Fig. 2). Like SMPDL3A, ASMase^cat^ possesses an additional C-terminal subdomain (CTD) consisting of four α-helices that pack up against the core burying 1,795 Å^2^ of surface area, thus distinguishing it from most other phosphoesterases. The linker connecting ASMase^cat^ to ASMase^sap^ is proline-rich and mostly rigid, wrapping itself around the catalytic domain as an L-shaped strap (Fig. 1b). The protein has five sites of N-glycosylation (three in ASMase^cat^, one in the linker and one in ASMase^sap^; Fig. 2c) and eight disulfide bonds (five in ASMase^cat^ and three in ASMase^sap^; Fig. 2a).

**Figure 3:**
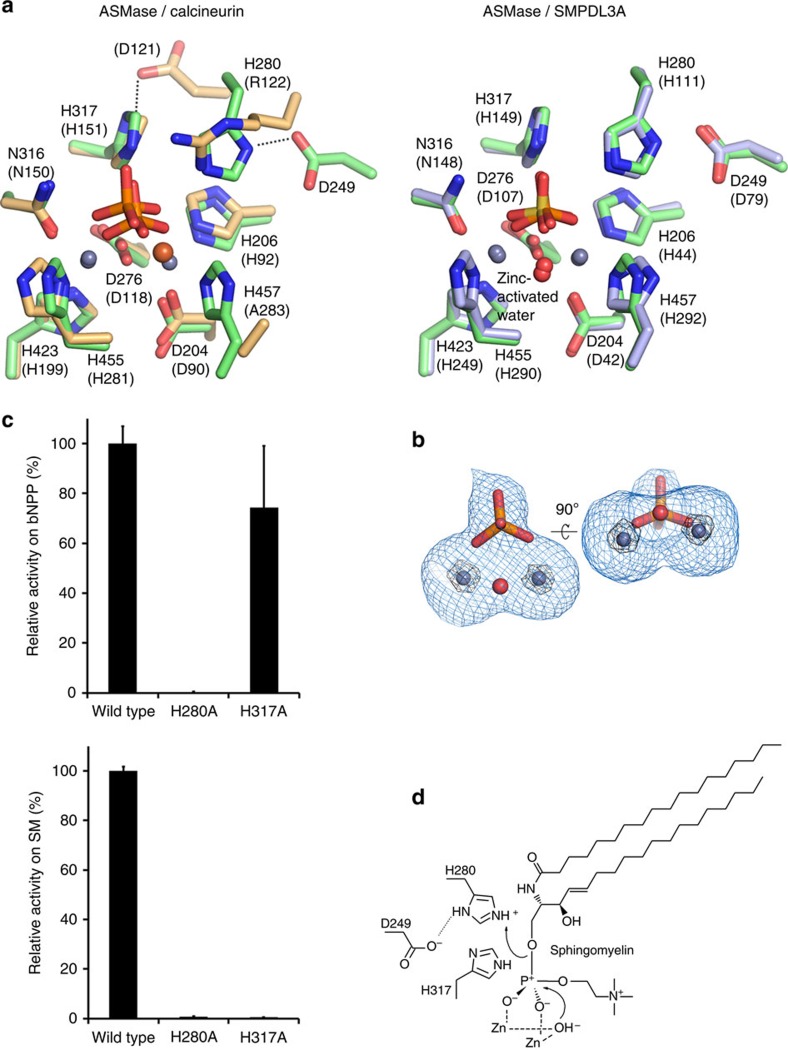
Comparison and mutation of the ASMase active site and proposed catalytic mechanism. (**a**) Active site of ASMase (green) is superimposed with that of calcineurin (PDB code 3LL8, beige) or SMPDL3A (PDB code 5FC1, blue). The active sites are very well conserved and likely use a similar mechanism of catalysis. However, there are slight differences. For example, in calcineurin the His–Asp proton-donating pair actually corresponds to His317 in ASMase, which is more likely involved in substrate binding. In addition, the His280 proton donor position is replaced with an arginine residue in calcineurin. (**b**) Electron density map around the zinc ions (blue spheres), bound phosphate and nucleophilic water (red sphere). Shown is an F_o_–F_c_ simulated annealing omit map contoured at 3*σ* (blue mesh) and 25*σ* (black mesh). The zinc-activated nucleophilic water molecule, which is part of the zinc octahedral coordination shell, could not be resolved separately in the electron density because of the resolution limit (2.6 Å). Thus, we based its position on the much higher resolution structure of SMPDL3A (PDB code 5FC1, 1.4 Å). (**c**) Top: substitution of His280 with alanine completely inactivates the enzyme, while the His317 to alanine substitution only slightly reduces activity against the non-lipid substrate bNPP. In absolute numbers, the activity of the wild-type enzyme is 1.76 μM bNPP hydrolyzed per nM protein per h. Data are the means and s.d.'s of two to five experiments performed in triplicates. Bottom: by contrast, using liposomes as substrate instead of bNPP, both His280 and His317 mutants abrogate enzymatic activity, suggesting that His317 is also important, probably for substrate binding or orientation. (**d**) The catalytic mechanism involves a nucleophilic attack by a zinc-activated water molecule on the phosphate group of sphingomyelin. This is followed by protonation of the ceramide leaving group by His280 with assistance from Asp249 and release of ceramide and phosphocholine.

Together, these elements form a spherical domain with a shallow depression on one side, at the base of which resides a two-zinc ion centre. In the structure of isolated ASMase^cat^, a phosphate ion is bound to the active site and forms several interactions with the protein and zinc ions, mimicking the scissile phosphate from sphingomyelin. As in other phosphoesterases the di-metal active site has octahedral coordination geometry from seven highly conserved protein residues, a water molecule and completed by the phosphate group (Figs 1c and 3a). In addition, His317 and His280 are positioned near the bound phosphate as potential proton donors for the ceramide leaving group (Fig. 1c). Mutation of these histidines and assessment of enzymatic activity at acidic pH with the generic small molecule phosphodiesterase substrate bis(*p*-nitrophenyl) phosphate (bNPP) revealed that His280 assisted by nearby Asp249 is the likely proton donor, while His317 is probably important for substrate binding (Fig. 3c). Together, these findings suggest that ASMase^cat^ catalyses sphingomyelin hydrolysis by the canonical mechanism used by phosphoesterases in which nucleophilic attack of a zinc-activated water molecule and protonation of the leaving group by His280 releases phosphocholine and ceramide (Fig. 3d).

### Interface between saposin and catalytic domains

**Figure 4:**
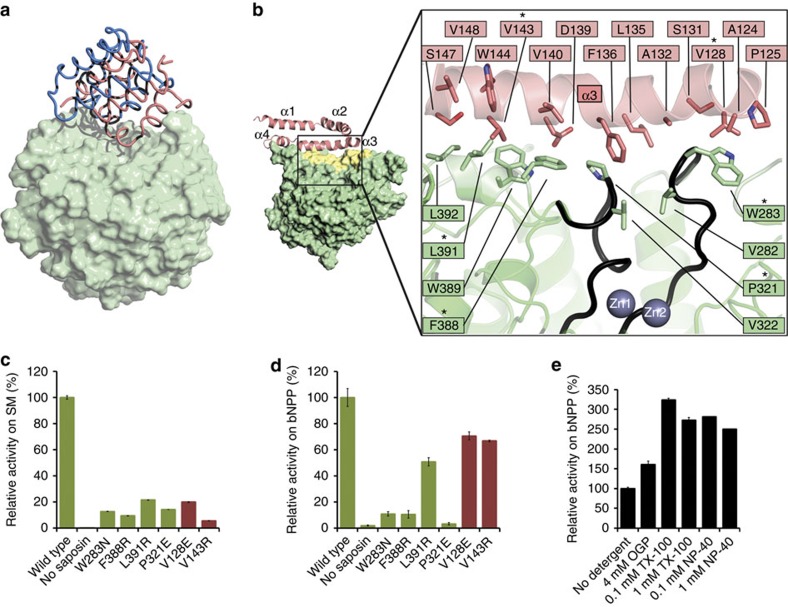
ASMase^sap^–ASMase^cat^ interactions. (**a**) Superposition of the two molecules in the asymmetric unit of the closed form. The reference portion for the superposition is the catalytic domain (light green surface). The saposin domains (worm representation) are shifted by roughly 8 Å, and the linker connecting the saposin domain to the catalytic domain is disordered in one of the molecules (blue). (**b**) Close-up of the interface formed between open ASMase^sap^ (pink) and ASMase^cat^ (green). Inset: yellow shading marks the surface buried on interface formation. Active site loops in contact with the interface are coloured black. Interface mutations tested are marked by an asterisk. (**c**) Activity at pH 5 of wild type (WT) and mutants on liposomes containing sphingomyelin (SM). Bar colours correspond to ASMase^cat^ (green) or ASMase^sap^ (pink) mutants. Activity is normalized to WT enzyme. 100% activity=537 μM SM hydrolyzed per μM protein per h on anionic liposomes. Data are the means and s.d.'s of two independent experiments performed in triplicates. (**d**) Activity at pH 5 of WT and mutants on the small-molecule substrate bNPP. Activity is normalized to the WT enzyme. 100% activity=1.76 μM bNPP hydrolyzed per nM protein per h. Data are from two to five experiments performed in triplicates. (**e**) Effect of detergents on activity against bNPP. Data are from two independent experiments performed in triplicates. OGP, octyl-β-D-glucopyranoside; NP-40, Tergitol-type NP-40; TX-100, Triton X-100. For the latter two detergents, 0.1 and 1 mM represent concentrations below and above their critical micellar concentrations.

ASMase^sap^ consists of four α-helices and belongs to the saposin family of proteins (saposins A, B, C and D)^21^. As observed with other saposins, ASMase^sap^ adopts either a closed or open conformation depending on environmental conditions (Fig. 1b). In the closed form, ASMase^sap^ is compact, globular and interacts mainly with the CTD (Fig. 1b), although this interaction appears to be a consequence of the crystal lattice since the two molecules in the asymmetric unit occupy slightly different positions, with the linker between the domains disordered in one molecule (Fig. 4a).

**Figure 5:**
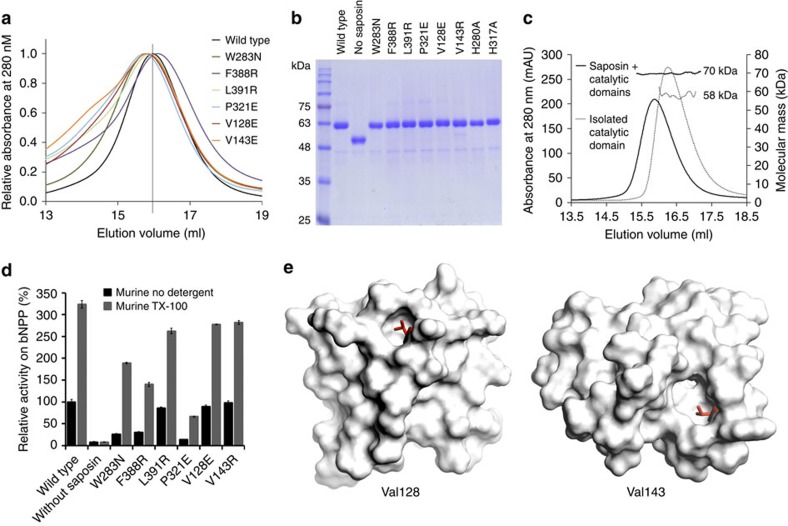
Purification of murine ASMase and bNPP hydrolysis assay. (**a**) Size-exclusion chromatography elution profiles of murine ASMase and point mutants. Ultraviolet absorbance is normalized. The elution volume of the wild-type protein (vertical marker) corresponds to a molecular weight 55 kDa, as extrapolated from a standard curve. (**b**) SDS–polyacrylamide gel electrophoresis of purified mutants. (**c**) Multi-angle light-scattering measurements of ASMase and the isolated catalytic domain. (**d**) Activity measurements of bNPP hydrolysis by ASMase interface mutants with added detergent (dark grey bars). 100% activity corresponds to 1.76 μM bNPP hydrolyzed per nM protein per h for ASMase. Addition of 0.2 mM Triton X-100 (TX-100) had no effect on the isolated catalytic domain. However, all interface mutants, which initially reduced enzymatic activity due to the mutation, had their activity restored to above wild-type levels. Data are the means and s.d.'s of triplicates. (**e**) We noted that the mutations on the saposin domain, V128E and V143R, had weaker inhibitory effects than those on the catalytic domain. This is likely because V128 and V143 are also important for stabilizing the closed conformation as shown (red sticks).

By contrast, the open form of ASMase^sap^ assumes a V-shaped conformation with its hydrophobic core exposed and its position shifted on ASMase^cat^ (Fig. 1b) such that one face of helix α3 forms an extensive 1,318 Å^2^, hydrophobic interface with ASMase^cat^ (Fig. 4b). To assess the significance of this interface for sphingomyelin hydrolysis, we measured the activity of interface mutants (Fig. 5a,b) on anionic liposomes containing sphingomyelin at acidic pH to mimic the lysosomal environment^21^. Deletion of ASMase^sap^ almost completely abrogated activity and six interface mutants substantially reduced activity, verifying that both the saposin domain and the interface are required for membrane sphingomyelin hydrolysis (Fig. 4c). The interface presumably stabilizes the open conformation of ASMase^sap^, allowing it to dock onto membrane surfaces, and possibly deliver lipids to the active site.

Closer examination of the interface revealed direct contacts between ASMase^sap^ and active site loops of ASMase^cat^ (Fig. 4b), hinting at a coupling between lipid binding, interface formation and catalytic activity. We assessed this hypothesis by reverting to the small-molecule bNPP substrate, which should depend only on catalytic domain attributes. Unexpectedly, we found that isolated ASMase^cat^ had no activity compared with wild type, and interface mutants also diminished activity on bNPP (Fig. 4d). Conversely, the addition of various detergents, which should bind to and favour open ASMase^sap^, substantially increased activity on bNPP (Fig. 4e), even in the context of the interface mutants (Fig. 5d). Analogous results were obtained for human ASMase (Supplementary Fig. 1). The structure of isolated ASMase^cat^ is unchanged relative to the catalytic domain in the open form full-length protein, but analysis of their crystal temperature factors indicated that interface formation reduces catalytic loop dynamics (Supplementary Fig. 2). This suggests that the interface is important not only for stabilizing open ASMase^sap^ for membrane docking, but also for stabilizing ASMase^cat^ and activating catalysis.

### Membrane docking

**Figure 6:**
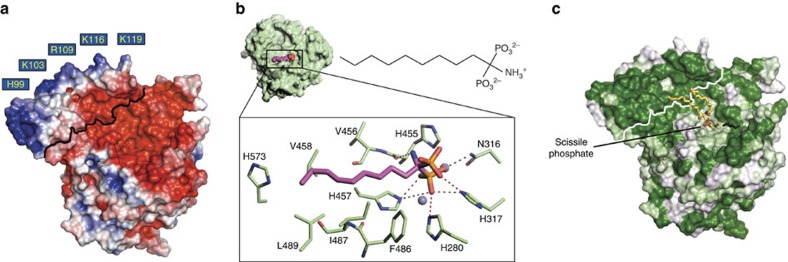
Electrostatic surface and substrate-binding site of ASMase. (**a**) Electrostatic surface representation of open ASMase contoured at ±3 kT. The calculation was done at pH 7 to highlight relative charge differences, which are otherwise masked at pH 5. Positively charged residues proposed to interact with anionic membrane lipids are labelled. A black line demarcates the ASMase^sap^:ASMase^cat^ boundary. A green outline marks the lipid-interacting surface on ASMase^sap^. (**b**) AbPA inhibitor bound to ASMase. Left: inhibitor shown as CPK spheres. Right: chemical structure of AbPA. Close-up, electrostatic interactions (red dashes) between AbPA (purple tail, orange and red phosphate) and zincs (spheres) and protein residues (green). (**c**) ASMase surface coloured by polar (white) to hydrophobic (dark green). A white line demarcates the ASMase^sap^:ASMase^cat^ boundary. Sphingomyelin (yellow sticks) was docked manually into the active site. For comparison, the end of the AbPA lipid tail is shown as black sticks.

The location on open ASMase^sap^ responsible for membrane lipid docking under physiological conditions likely includes its hydrophobic surface, which in the crystal is buried by a symmetry-related saposin domain reminiscent of the ‘handshake' clasp observed in saposin B dimers^12^ (Supplementary Fig. 3a). Indeed, crystallization in the presence of lipids rearranges the saposin dimer and reveals lipid molecules bound within it (Supplementary Fig. 3b). In addition, the top edge of helix α2, which is lined with a ridge of positively charged residues, is appropriate for interaction with anionic intra-lysosomal vesicles^21^ (Fig. 6a). Consistent with this, mutation of Lys120 (murine Lys116) to glutamate decreases ASMase activity^22^, and CADs interfere with ASMase membrane attachment, thereby indirectly inhibiting its activity^23^. Accordingly, inclusion of CADs in our liposomal assay markedly reduced ASMase activity (Supplementary Fig. 4), presumably by blocking the electrostatic interaction between helix α2 and the anionic liposomes. CADs are critical for the treatment of patients with major depressive disorder where ASMase is hyperactive^5^, and can kill several types of cancer cells where ASMase activity is already debilitated^6^. However, their indirect mechanism of action makes them promiscuous and consequently, it is imperative to develop direct ASMase inhibitors with better selectivity.

### Inhibitor-bound ASMase

To further these efforts, we determined the structure of ASMase bound to a known high-affinity inhibitor, 1-aminodecylidene bis-phosphonic acid (AbPA; half-maximal inhibitory concentration 20 nM)^24^ (Fig. 6b and Supplementary Fig. 5). One phosphate group of AbPA completes the coordination shell for the zinc ions as described above, whereas its nine-carbon lipid tail sits in a relatively featureless shallow groove. This structure and the position of the leaving group-protonating His280 allowed us to propose a model for how sphingomyelin might bind to the active site (Fig. 6c). The fatty-acid chains of sphingomyelin sit along a hydrophobic track that extends from the edge of the active site to the saposin domain and surprisingly, do not correspond to the position of the AbPA lipid tail, which instead coincides with the position of the choline head group. These results will help direct the rational design of new ASMase inhibitors that better exploit the shape of the active site.

### Structural mapping of Niemann–Pick mutations

**Figure 7:**
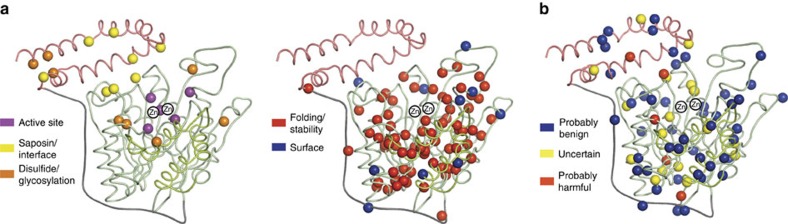
Structural mapping of disease mutations. (**a**) Mapping of Niemann–Pick mutations on ASMase. Mutations are indicated by spheres coloured according to their predicted effect. (**b**) Locations of additional ASMase variants of unknown significance.

ASMase is best known for its causative role in Niemann–Pick disease types A and B. Over 130 missense mutations and short indels have been identified in the ASMase gene of patients suffering from this autosomal recessive disease. According to our structure, 103 mutations reside in the catalytic core, 19 in the CTD, 8 in the saposin domain and 4 in the Pro-rich linker (Fig. 7a). Most mutations are predicted to affect the fold or stability of ASMase (Supplementary Table 1). However, about 12 are surface mutations that could affect the interaction of ASMase with membranes or other proteins, and 5 are located in the ASMase^sap^–ASMase^cat^ interface, which could impair the regulatory mechanism described here. Interestingly, 80 ASMase variants of unknown significance have also been reported^3^. Half of these are surface exposed with no obvious deleterious effects; however, several are predicted to significantly affect the fold or stability of ASMase (Fig. 7b and Supplementary Table 2). Further characterization of these potentially harmful mutations will have important implications for Niemann–Pick carrier screening programmes^25^ on higher-risk populations.

## Discussion

**Figure 8:**
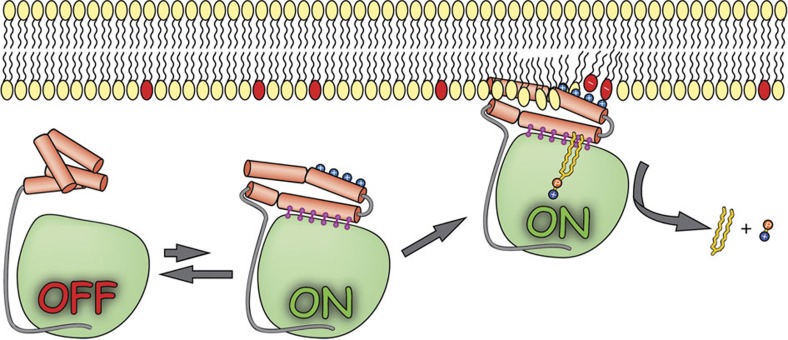
Schematic for ASMase activation. Membrane lipids that have red head groups indicate negative charges. Released products are ceramide (yellow worm) and phosphocholine (orange and blue circles).

Taken together, we propose a model whereby ASMase in solution exists in equilibrium between open and closed forms of the saposin domain (Fig. 8). In the absence of membranes, closed ASMase^sap^ decoupled from ASMase^cat^ would predominate and render the enzyme inactive. In the presence of anionic membranes, open ASMase^sap^ becomes prevalent, docks onto the membrane surface and concomitantly forms an interface with the catalytic domain activating it for sphingomyelin hydrolysis.

Our structural analysis of ASMase is an important step towards rationally designed ASMase inhibitors for treating the myriad of pathologies this enzyme mediates, predicting the effects of newly discovered mutations, as well as developing recombinant ASMase replacement therapy for hereditary diseases^1^.

## Methods

### Constructs

ASMase was cloned into a derivative of pFastBac 1 (Invitrogen). The vector contained the cleavable melittin signal peptide MKFLVNVALVFMVVYISYIYA followed by a hexahistidine tag DRHHHHHHKL. Constructs of human (RefSeq NP_000534.3) and murine (RefSeq NP_035551.1) ASMase extended from residues 84 to 611 and 88 to 615, respectively. The crystallized construct of the isolated murine ASMase catalytic domain extended from residues 165 to 627. For activity assays, isolated catalytic domain constructs encompassed residues 169–615 of human ASMase and 165–611 of its murine counterpart. All constructs and mutants were sequenced.

### Protein expression and purification

Recombinant baculoviruses were generated according to the Bac-to-Bac Baculovirus Expression System protocol (Invitrogen) with minor modifications: DH10MultiBac cells were used^26^ and viruses were added to *Sf9* cells (Invitrogen) grown in I-Max medium (Wisent Bioproducts). Proteins were expressed at 27 °C for 64 h. Subsequent steps were carried out at 4 °C. Cells were removed by centrifugation at 1,000*g* then at 9,000*g*, and supernatants were incubated with HisPur Ni-NTA resin (Thermo Fisher Scientific). The beads were washed with buffer A (50 mM Tris-HCl (pH 7.5), 500 mM NaCl and 1 mM MgCl_2_) and eluted with buffer A containing 250 mM imidazole-HCl. Proteins were concentrated and loaded on a Superdex 200 10/300 GL size-exclusion column (GE Healthcare) equilibrated with buffer B (15 mM Tris-HCl (pH 7.5), 100 mM NaCl and 10 μM ZnCl_2_). Fractions containing ASMase were applied to a Mono Q anion-exchange column (GE Healthcare) in buffer B. The flow-through was concentrated to 10 mg ml^−1^ and flash-frozen.

### Enzymatic assay with non-lipid substrate

Protein at 100 nM (1 μM for the H280A mutant) was incubated with 2 mM bis(*p*-nitrophenyl) phosphate in assay buffer (100 mM NaCl and 20 mM sodium acetate (pH 5)) at 37 °C for 1 h. NaOH was then added to a final concentration of 100 mM before measuring absorbance at 405 nm. Activity was quantified by a *p*-nitrophenol standard curve.

### Liposome-based enzymatic assay

Liposomes were prepared by extrusion through 0.1-μm polycarbonate filters. Negatively charged liposomes consisted of 10% egg sphingomyelin, 55% dioleoylphosphatidylcholine, 20% cholesterol and 15% bis(monooleoylglycero)phosphate (BMP). The Amplex Red Sphingomyelinase Assay (Thermo Fisher Scientific) for phosphocholine detection was slightly modified. ASMase at 100 to 1,000 nM was incubated with liposomes at 3 mM total lipids in assay buffer for 1 h at 37 °C. The reaction was terminated at 95 °C for 5 min and an equal volume of the second-step solution was added as recommended. The second step was carried out at 37 °C and the change in fluorescence (560 nm excitation and 590 nm emission) after 30 min was used to quantify product formation with the help of a phosphocholine standard curve.

### Crystallization and data collection

Crystals were grown by sitting drop or hanging drop vapour diffusion at 22 °C. Protein at 10 mg ml^−1^ in buffer B was mixed with an equal volume of well solution. Crystals of murine ASMase with the saposin domain in an open conformation (∼200 × 25 × 25 μm in size) were obtained in 100 mM sodium MES (pH 6.5) and 1.5 M ammonium sulfate. The same protein with the saposin domain in a closed conformation was crystallized (50 × 50 × 50 μm) in 0.2 M lithium acetate and 20% PEG 3350 by first incubating it with 5 mM AbPA (Cayman Chemical) at 22 °C for 12 h. The protein was also incubated with Triton X-100 and sphingomyelin or octadecylphosphonic acid (Sigma-Aldrich) at 22 °C for 12 h, and crystals with the saposin domain in a slightly different open conformation (100 × 100 × 25 μm) were grown in 100 mM NaH_2_PO_4_, 100 mM KH_2_PO_4_, 100 mM sodium HEPES (pH 6.5) and 2 M NaCl. The same crystallization condition also yielded crystals of the isolated catalytic domain (25 × 25 × 25 μm). All crystals were briefly soaked in well solution supplemented with 20% glycerol before flash-freezing. Diffraction data were collected at 100 K on beamlines 08ID-1 equipped with a Rayonix MX300 CCD detector at a wavelength of 0.97949 Å (beam size of 130 × 30 μm), or 08B1-1 with a Rayonix MX300HE CCD detector at 1.28154 Å (230 × 195 μm) in the case of the AbPA-bound crystal, at the Canadian Macromolecular Crystallography Facility, Canadian Light Source. Data collection parameters were as follows: for the open conformation crystal, 1 s 0.45° images covering 119.7°; for the AbPA-bound crystal, 15 s 0.5° images covering 270°; for the open conformation crystal in the presence of lipid, 1 s 0.25° images covering 180°; for the catalytic domain crystal, 2 s 0.85° images covering 378.25°. Data was processed with HKL2000 (ref. 27) or XDS^28^.

### Structure determination and refinement

All structures of ASMase were solved with the Phaser/Phenix^29^ molecular replacement package using a 1.4-Å resolution experimentally determined structure of the ASMase paralogue, SMPDL3A lacking the saposin domain (PDB code 5FC1). After rebuilding in Coot^30^ (Supplementary Fig. 6) and refinement in Phenix^29^ using metal coordination restraints but no non-crystallographic symmetry restraints, the saposin domain could be located and built from difference electron density. The crystal of ASMase bound to octadecylphosphonic acid had a twin fraction of 50% and was refined with the twin law h,-k,-l. Structural images were generated with PyMOL (The PyMOL Molecular Graphics System, Version 1.3 Schrödinger, LLC).

### Data availability

Coordinates and structure factors have been deposited in the Protein Data Bank under accession codes 5FI9 for the closed form of the saposin domain, 5FIB for the open form, 5FIC for the open form in presence of lipid and 5HQN for the isolated catalytic domain. All other data are available from the authors on request.

## Additional information

**How to cite this article:** Gorelik, A. *et al*. Crystal structure of mammalian acid sphingomyelinase. *Nat. Commun.* 7:12196 doi: 10.1038/ncomms12196 (2016).

## Supplementary Material

Supplementary Information Supplementary Figures 1-6 and Supplementary Tables 1-2

Peer review file

## Figures and Tables

**Table 1:** X-ray data collection and refinement statistics.

**Crystal**	**Closed form with AbPA**	**Open form**	**Open form in presence of lipid**	**Catalytic domain**
PDB code	5FI9	5FIB	5FIC	5HQN
				
*Data collection*				
Space group	P1	P42_1_2	P4_1_	C2
Cell dimensions				
*a*, *b*, *c* (Å)	57.39, 72.02, 74.42	181.17, 181.17, 109.90	101.75, 101.75, 401.83	124.84 127.30 102.52
*α*, *β*, *γ* (°)	80.47, 71.52, 83.60	90, 90, 90	90, 90, 90	90, 121.68, 90
Resolution (Å)	46.65–2.54 (2.63–2.54)	45.70–2.80 (2.90–2.80)	49.32–2.80 (2.90–2.80)	37.67–2.60 (2.69–2.60)
*R*_meas_	0.12 (0.67)	0.13 (1.62)	0.21 (1.26)	0.19 (2.00)
*I*/*σI*	9.7 (1.6)	17.3 (1.7)	10.2 (1.46)	10.0 (1.44)
Completeness (%)	97.2 (90.9)	100 (100)	100 (100)	100 (100)
Redundancy	2.9 (2.5)	9.7 (9.0)	6.7 (4.9)	7.7 (6.5)
Wilson *B*-factor (Å^2^)	38.0	40.7	48.4	52.9
				
*Refinement*				
Protein molecules per ASU	2	2	4	2
Resolution (Å)	46.65–2.54	45.70–2.80	49.32–2.80	37.67–2.60
Number of unique reflections	33,629	39,766	99,738	41,929
*R*_work_/*R*_free_	20.2/25.4	18.8/23.9	19.0/23.9	21.3/25.8
Number of atoms				
Protein	8,294	8,474	16,675	7,050
Glycans, ligands and ions	410	521	286	226
Water	54	30	39	74
*B*-factors (Å^2^)				
Protein	41.7	41.9	57.5	51.2
Glycans, ligands and ions	64.4	96.2	76,1	88.8
Water	29.6	25.4	31.6	44.5
Ramachandran statistics				
Favoured (%)	93.3	94.0	95	94.5
Allowed (%)	6.6	5.8	4.9	5.5
Outliers (%)	0.1	0.2	0.1	0.0
Rotamer outliers (%)	0.8	0.6	0.4	0.8
R.m.s. deviations				
Bond lengths (Å)	0.003	0.003	0.004	0.005
Bond angles (°)	0.72	0.82	0.72	0.67

ASU, asymmetric unit; R.m.s., root mean squared.

Values for the highest-resolution shell are shown in parentheses.
