# Supplementary material for: Crystal structure of mammalian acid sphingomyelinase
Source: Nat Commun. 2016 Jul 20;7:12196. doi: 10.1038/ncomms12196 (PMC4961792; doi:10.1038/ncomms12196)
Supplement: Supplementary Information — Supplementary Figures 1-6 and Supplementary Tables 1-2 [file ncomms12196-s1.pdf]

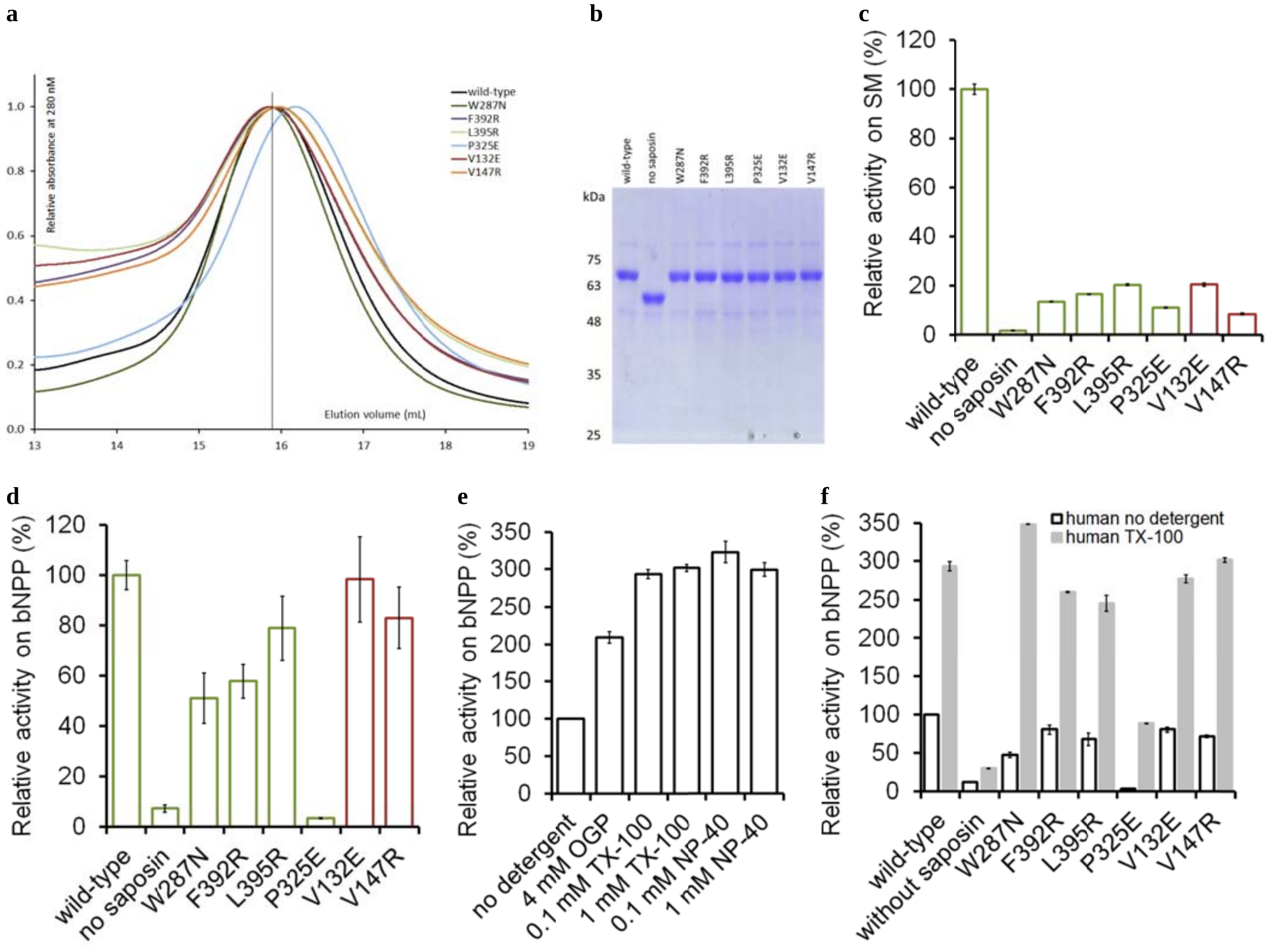

Supplementary Figure 1 | Purification of human ASMase and activity assays

(a) Size exclusion chromatography elution profiles of human ASMase and point mutants. UV absorbance is normalized. The elution volume of the wild-type protein (vertical marker) corresponds to a molecular weight of 57 kDa, as extrapolated from a standard curve. **(b)** SDS-PAGE of purified mutants. **(c)** Activity measurements of wild-type and mutant proteins on liposomes containing sphingomyelin (SM). Activity is normalized to the wild-type enzyme. 100% activity corresponds to 534 µM SM hydrolyzed per µM protein per hour on anionic liposomes. Data are representative of two independent experiments performed in triplicates. **(d)** Activity measurements of wild-type and mutant proteins on the small molecule substrate bis(p-nitrophenyl) phosphate (bNPP). Activity is normalized to the wild-type enzyme. 100% activity corresponds to 1.11 µM bNPP hydrolyzed per nM protein per hour. Data are the means and standard deviations of two to five experiments performed in triplicates. **(e)** Effect of detergents on activity of wild-type protein against the non-lipid substrate bNPP. Data are representative of two independent experiments performed in triplicates. OGP, octyl β-D-glucopyranoside. TX-100, Triton X-100. NP-40, Tergitol type NP-40. For the latter two detergents, 0.1 mM and 1 mM represent concentration below and above their critical micellar concentrations. **(f)** Activity measurements of bNPP hydrolysis by ASMase interface mutants with added detergent. Data are the means of triplicates. In all panels, error bars represent the standard deviation.

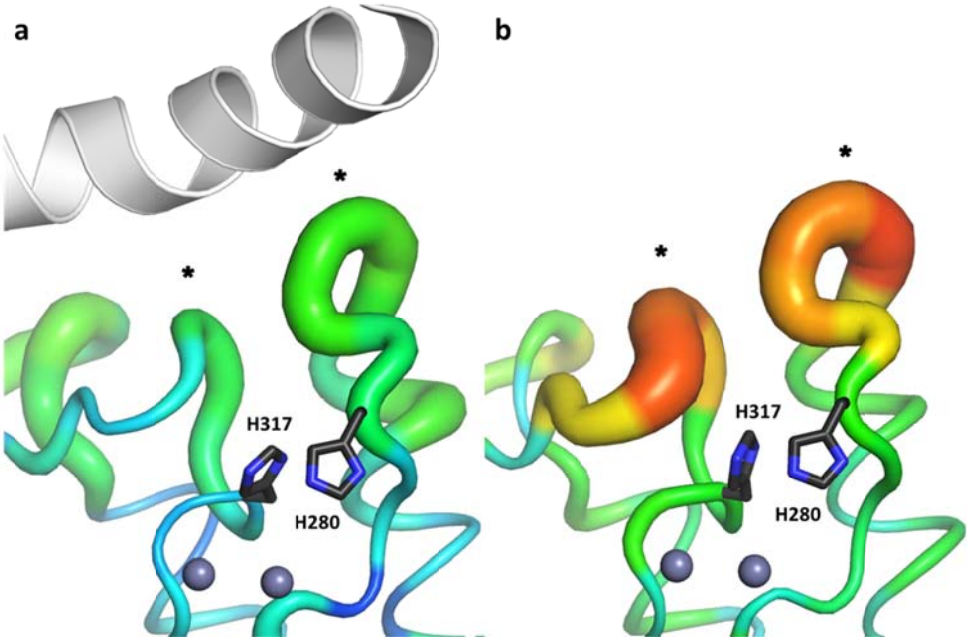

Supplementary Figure 2 | Main chain B-factor analysis of catalytic domain interface loops

The two histidines of the active site are displayed as sticks, and the loops at the inter-domain interface leading to these histidines are marked with asterisks. **(a)** In the structure with the open form of the saposin domain, catalytic domain loops have relatively lower B-factors due to the interactions with the saposin domain (white helix). **(b)** In the structure of the isolated catalytic domain, B-factors are relatively higher since the interface is absent. Low temperature factors are colored in blue, while high temperature factors, indicative of relative higher mobility, are shown in red. Note that the color and thickness are for the relative B-factors within each structure, and not absolute B-factor values across both structures. The range of α-carbon B-factors is 21 to 50 Å^2 in (a) and 37 to 83 Å^2 in (b).

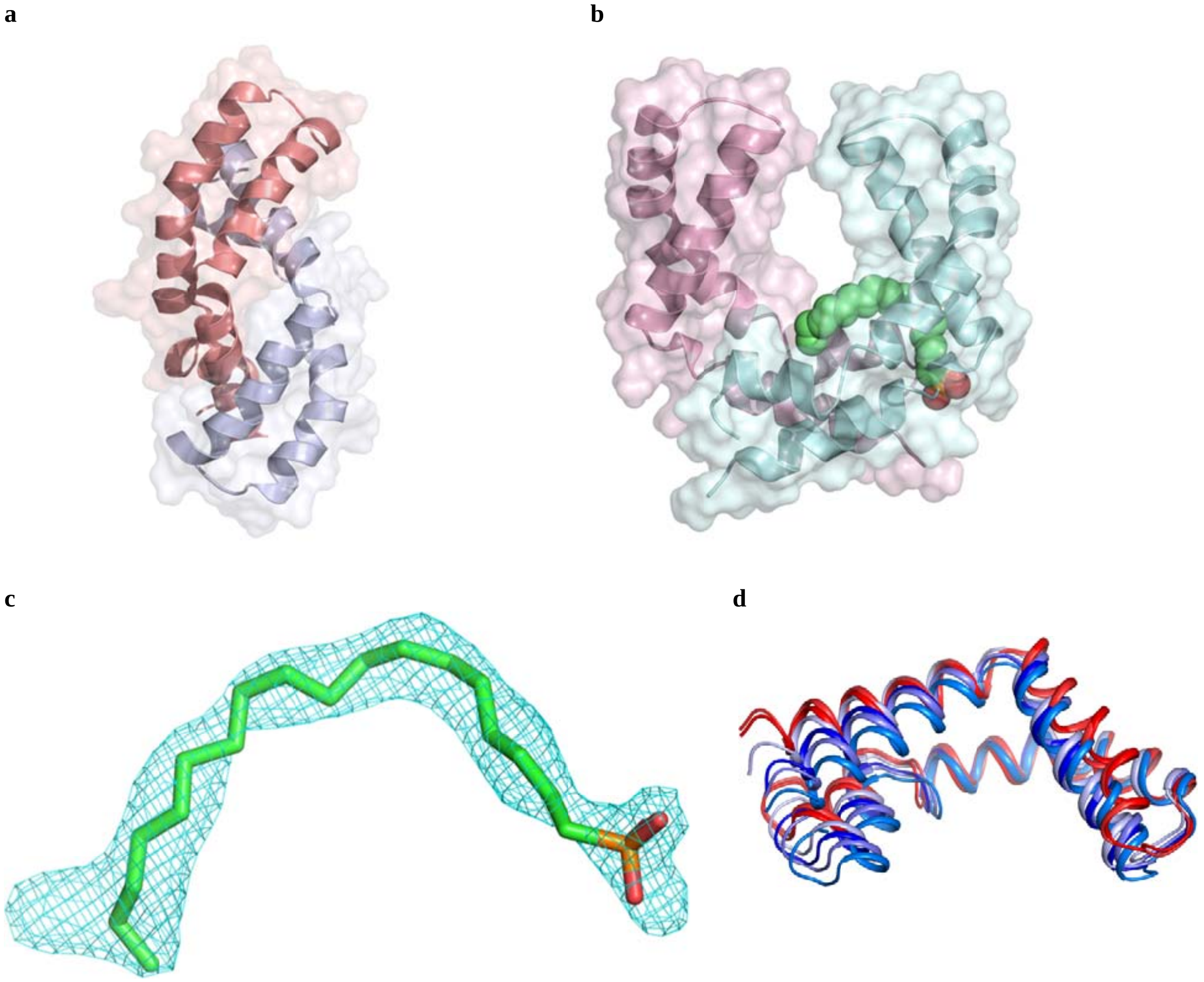

Supplementary Figure 3 | Saposin dimers in the crystal lattice

(a) Rearrangement to the V-shaped conformation of ASMase^{sap} exposes its hydrophobic core, which in the crystal is buried by a symmetric dimerization with another saposin domain. **(b)** Co-crystallization in the presence of lipid causes the dimer to shift such that a cavity is created at its center, and a molecule of octadecylphosphonic acid (ODPA) is bound between the saposin monomers. **(c)** F_o-F_c electron density map contoured at 2.5 σ before inclusion of the lipid molecule. Density for a second lipid molecule is also present within the dimer; however, it is fragmented and its last carbon atom overlaps with density for the first molecule, indicating likely partial occupancy of the lipid molecules in different positions. **(d)** Comparison of the saposin domains from the four protein chains of the lipid-bound structure (blue) and from the two chains of the apo structure (red). Proteins were superimposed via their catalytic domain (not shown).

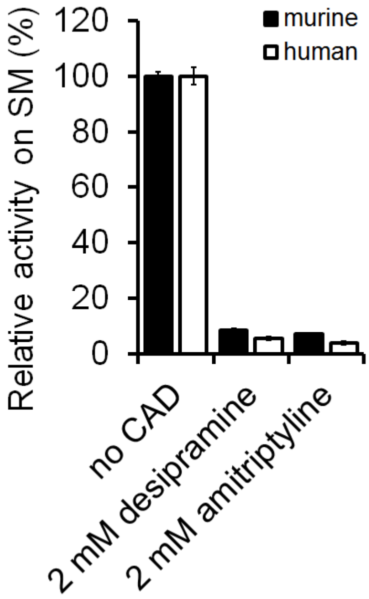

Supplementary Figure 4 | Liposomal activity assay of ASMase in the presence of cationic amphiphilic drugs (CADs). Error bars represent the standard deviation of triplicates.

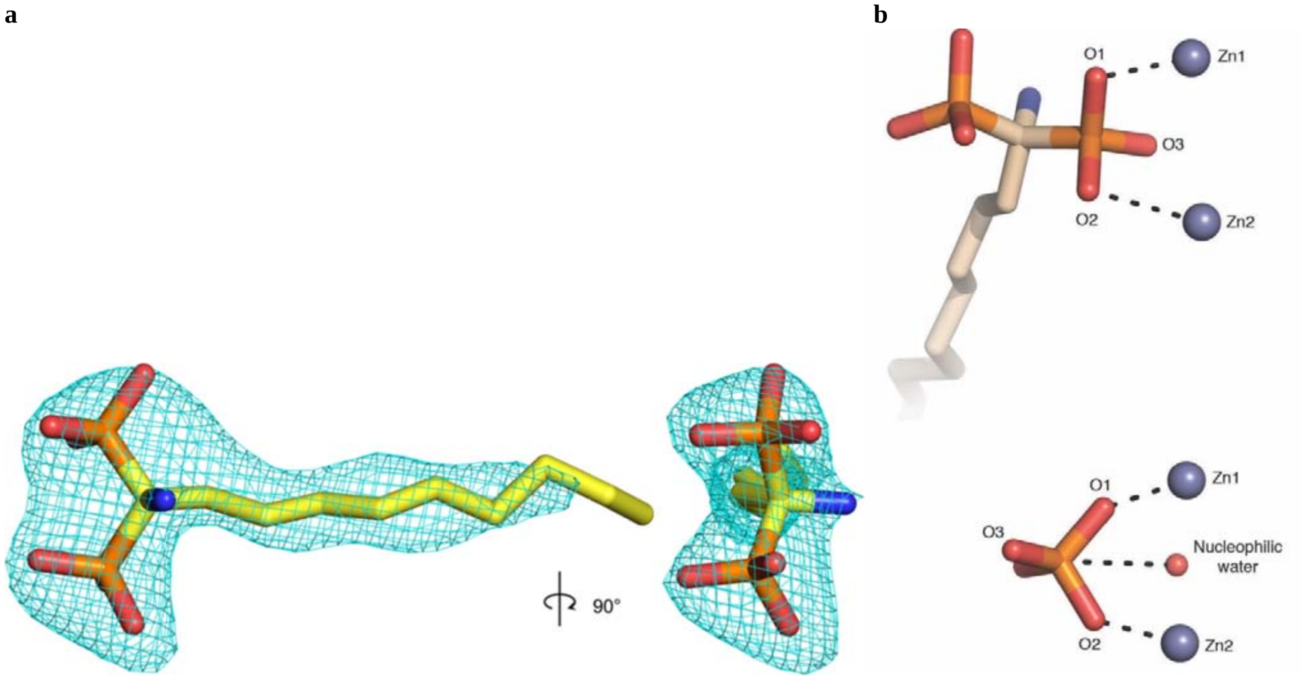

Supplementary Figure 5 | Electron density and binding mechanism for the co-crystallized inhibitor AbPA

(a) Simulated annealing omit F_o-F_c electron density map contoured at 3 σ. **(b)** Comparison of the phosphate-bound active site of ASMase (below) vs that of AbPA-bound (above). Interestingly, in the AbPA-bound structure the phosphate group appears to be positioned as a product as if its configuration has been inverted after nucleophilic attack by the zinc-activated water molecule. Thus for AbPA, the phosphate group not only blocks the substrate binding site, but also completes the octahedral zinc coordination shell by excluding the nucleophilic water molecule. In the phosphate-bound structure, the phosphate group is positioned as a substrate with O3 pointed in a direction opposite to that of AbPA. The structures are in the identical orientation to illustrate the difference in the phosphate group between the two ligands. Zinc ions and the nucleophilic water are labeled.

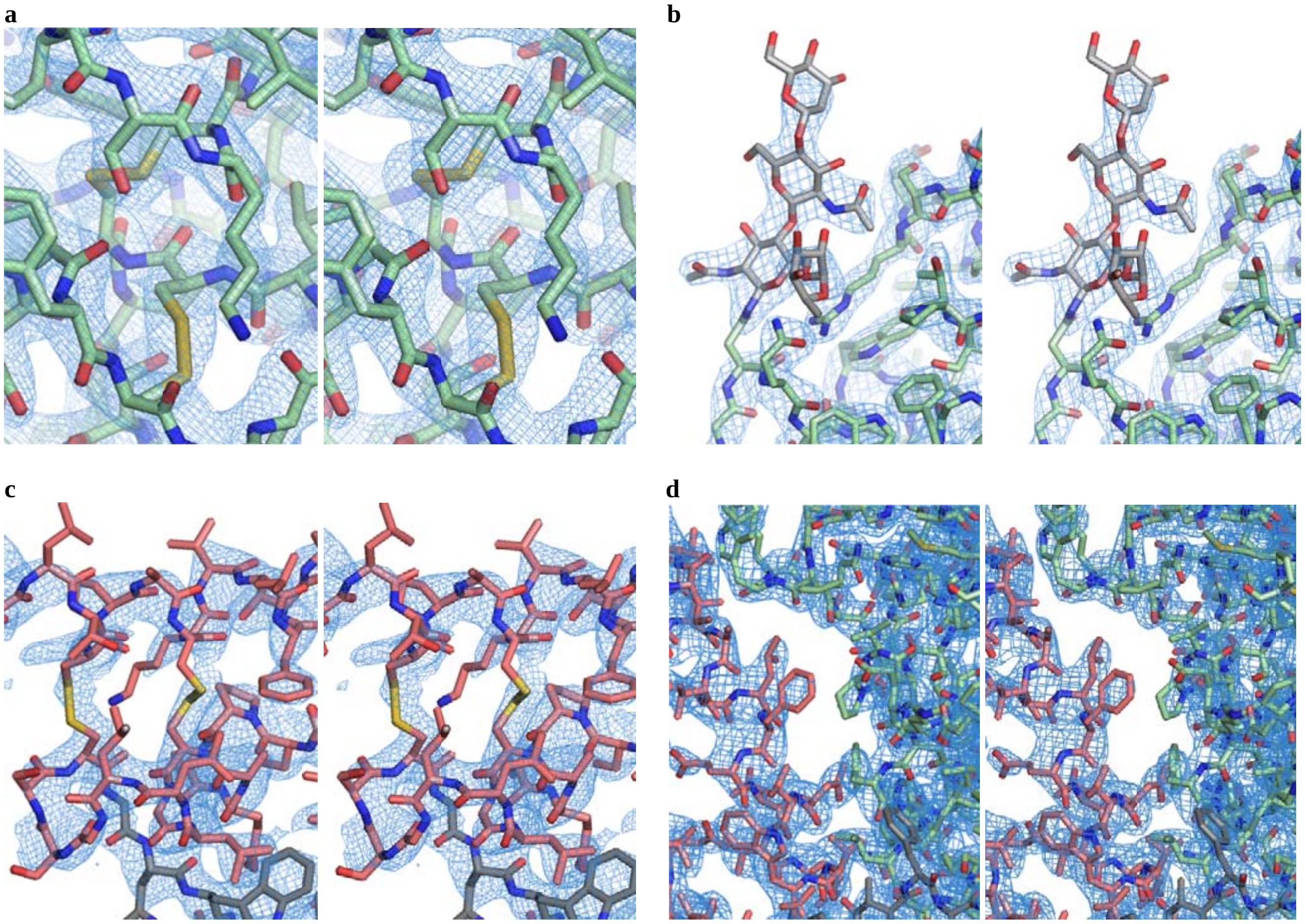

Supplementary Figure 6 | Representative electron density maps of the ASMase structures

A wall-eyed stereo view of the $2F_o$-F_c electron density maps, contoured at 1.5 σ. **(a)** Isolated catalytic domain structure showing a disulfide-rich loop. **(b)** A glycan on the structure with the saposin domain in a closed form. **(c)** The open form of the saposin domain; the latter is stabilized by disulfide bonds. **(d)** The inter-domain interface in the structure with the saposin domain in an open form in the presence of lipid.

Mutations that disrupt the active site							
H321Y	D280A	H427R	H461P	D253E	D253H		
Mutations that alter the saposin domain hydrophobic surface or its interface with the catalytic domain							
L105P	V114M	L163P	F392del	V132A	L139P	P325A	W393G
Mutations that prevent disulfide bond formation or glycosylation							
C91H	C94W	C159R	C228R	C387R	C433R	C596Y	N522S
N522D							
Mutations predicted to affect the folding or stability of the protein							
F482del	T594del	R610del	G168R	I178N	A198P	R202C	W211R
L227M	L227P	R230C	R230H	A243V	G244R	W246C	G247D
G247S	S250R	T258I	A283T	R291H	Q294K	L304P	V314M
Y315H	P332R	W342C	L343P	A359D	A359V	Y369C	P373S
R378H	R378L	S381F	S381P	L382F	M384I	N385K	N385S
N391T	A415V	H423R	H423Y	P429L	P430S	L434P	W437C
W437R	S438R	Y448C	T451P	L452P	A453D	(G)A454V	G458D
T460P	F465S	Y469S	R476W	R476Q	P477L	F482L	F482S
A484E	A484V	S486R	T488A	(F)Y490N	G496C	G496S	R498C
R498H	R498L	Y500H	H516Q	E517V	Y519C	P533L	W535R
Y539H	A541T	(M)L551P	V559L	F572L	H577L	H577R	H577D
K578N	G579S	Q598R	L599F	R602H	R602P	S436_W437dup	
Surface mutations without clear deleterious effect							
P186L	G234D	E248K	E248Q	R296Q	G336S	E471K	(N)G492S
Q525H	H556Y	D565Y	R610C				

Supplementary Table 1 | Predicted effects of ASMase mutations found in Niemann-Pick patients

Mutations were classified according to their predicted effect, based on the ASMase structure. When the human and murine amino acid sequences differ, the latter is indicated in parentheses.

**Variants that prevent disulfide bond formation or glycosylation**							
Probably harmful:		C433W	(P)S507F				
**Variants that alter the saposin domain hydrophobic and positively charged surfaces or its interface with the catalytic domain**							
Probably benign:		(L)I101V	V117M	(A)I136V	R113H		
Uncertain:		A158T	R113C	V318E	T324I	V132M	
Probably harmful:		V145del	P325delinsLS				
**Other non-surface variants**							
Probably benign:		N102S	G115S	L256P	G270S	(I)V301I	P331S
A346V	(R)Y374F	A446V	N450S	A487V*	Y519F	I520V	(A)T550A
V559I	R591H	L599I					
Uncertain:		L266V	A269V	P282T	R296W	A297V	V305M
P332L	R378C	V409G	P430A	E449Q	(G)A454S	V512M	R591C
Probably harmful:		L379F	S510F				
**Surface variants, probably benign**							
S192del	G104R	R150C	(T)S175P	P187S	(Q)R240Q	(F)Y245H	(Q)H288D
(S)T290N	(D)A303T	P313S	(Q)R341C	(Q)R341H	(Q)R341P	E352D	(H)R361C
(H)R361H	R389C	(K)R443G	(K)R443Q	E472D	G508R*	G530A	R542Q
N557K	R561H	D565N	P582S	S583L	T588M	R610H	M613I

Supplementary Table 2 | Predicted effects of ASMase variants of unknown significance

Variants were classified according to their predicted effect, based on the ASMase structure. Reported polymorphisms are marked by an asterisk. When the human and murine amino acid sequences differ, the latter is indicated in parentheses.
