# Supplementary material for: Crystal structure of mammalian acid sphingomyelinase
Source: Nat Commun. 2016 Jul 20;7:12196. doi: 10.1038/ncomms12196 (PMC4961792; doi:10.1038/ncomms12196)
Supplement: Peer review file [file ncomms12196-s2.pdf]

Editorial Note: this manuscript has been previously reviewed at another journal that is not operating a transparent peer review scheme. This document only contains reviewer comments and rebuttal letters for versions considered at Nature Communications.

REVIEWERS' COMMENTS:

Reviewer #1 (Remarks to the Author):

The authors have addressed all my previous comments satisfactory and the revised manuscript is greatly improved. I don't have any additional questions, except for couple remarks:

1. SAXS data have been removed from the text and figures, however the description of SAXS experiments still remains in the methods. It should be removed as well.

2. Regarding crystallographic data collection details, I didn't mean adding them to Table 1. They are typically provided in the methods. But I don't have any problem with the way how they are presented in the table in the current version, if it complies with the journal style. Please, mention what detector was used for data collection.
